# Human Solid Tumor Xenografts in Immunodeficient Mice Are Vulnerable to Lymphomagenesis Associated with Epstein-Barr Virus

**DOI:** 10.1371/journal.pone.0039294

**Published:** 2012-06-18

**Authors:** Kui Chen, Sharif Ahmed, Oyedele Adeyi, John E. Dick, Anand Ghanekar

**Affiliations:** 1 Toronto General Research Institute, University Health Network, Toronto, Ontario, Canada; 2 Department of Pathology, University Health Network, Toronto, Ontario, Canada; 3 Division of Stem Cell and Developmental Biology, Campbell Family Institute for Cancer Research/Ontario Cancer Institute, Toronto, Ontario, Canada; 4 Department of Molecular Genetics, University of Toronto, Toronto, Ontario, Canada; 5 Department of Surgery, University of Toronto, Toronto, Ontario, Canada; Duke University Medical Center, United States of America

## Abstract

Xenografting primary human solid tumor tissue into immunodeficient mice is a widely used tool in studies of human cancer biology; however, care must be taken to prove that the tumors obtained recapitulate parent tissue. We xenografted primary human hepatocellular carcinoma (HCC) tumor fragments or bulk tumor cell suspensions into immunodeficient mice. We unexpectedly observed that 11 of 21 xenografts generated from 16 independent patient samples resembled lymphoid neoplasms rather than HCC. Immunohistochemistry and flow cytometry analyses revealed that the lymphoid neoplasms were comprised of cells expressing human CD45 and CD19/20, consistent with human B lymphocytes. *In situ* hybridization was strongly positive for Epstein-Barr virus (EBV) encoded RNA. Genomic analysis revealed unique monoclonal or oligoclonal immunoglobulin heavy chain gene rearrangements in each B-cell neoplasm. These data demonstrate that the lymphoid neoplasms were EBV-associated human B-cell lymphomas. Analogous to EBV-associated lymphoproliferative disorders in immunocompromised humans, the human lymphomas in these HCC xenografts likely developed from reactivation of latent EBV in intratumoral passenger B lymphocytes following their xenotransplantation into immunodeficient recipient mice. Given the high prevalence of latent EBV infection in humans and the universal presence of B lymphocytes in solid tumors, this potentially confounding process represents an important pitfall of human solid tumor xenografting. This phenomenon can be recognized and avoided by routine phenotyping of primary tumors and xenografts with human leukocyte markers, and provides a compelling biological rationale for exclusion of these cells from human solid tumor xenotransplantation assays.

## Introduction

Xenotransplantation of human cancers into immunodeficient mice is very useful for studying human tumor biology [1]. This approach is widely used for research into mechanisms of tumor growth and for preclinical evaluation of anti-cancer therapies [2], [3]. The most commonly utilized mouse strains, such as the non-obese diabetic severe combined immunodeficiency (NOD/SCID) and NOD/SCID/interleukin 2 receptor gamma chain null (NSG) strains, are deficient in both innate and adaptive immunity and thereby permit the survival of human tissue [4], [5].

While it is possible to generate xenografts from purified populations of cells from some human cancers [6]–[9], many studies describe the implantation of tumor fragments or bulk tumor cell suspensions in mice to maximize the chances of establishing xenografts from limited clinical samples [10]–[13]. Reports describing xenografts from human hepatocellular carcinoma (HCC), for example, reveal that only 10–20% of clinical samples yielded viable xenografts when dissociated into cell suspensions that were further fractionated for implantation in mice [14], [15].

Epstein-Barr virus (EBV) is a human-restricted herpes virus which infects over 90% of the human population, persisting as a latent infection for the lifetime of the host [16], [17]. EBV preferentially infects B lymphocytes and “transforms” them into a proliferative state by altering cellular gene transcription, constitutively activating key cell-signalling pathways, and preventing apoptosis [18]. In immunocompetent individuals, EBV is well controlled by cellular and humoral immunity, and transformed B cells are continually eliminated because they express foreign antigens. Individuals who are immunocompromised, such as patients with HIV/AIDS or those receiving immunosuppressive drugs following transplantation, are at risk of developing B-cell lymphomas from the uncontrolled proliferation of EBV-transformed cells [17], [19], [20]. Similarly, spontaneous development of EBV-associated human lymphomas has been described in immunodeficient mice repopulated with normal human hematopoietic cells due to reactivation of latent EBV [21].

In our attempts to efficiently generate xenografts from human HCC specimens, we implanted tumor fragments or bulk tumor cell suspensions into immunodeficient mice. To validate our model, we carefully examined xenografts to determine how accurately they recapitulated parent tumors. Unexpectedly, we observed that several xenografts did not resemble HCC, and sought to characterize these tumors further in order to understand potential pitfalls of our xenotransplantation assay. In this report, we describe how our further characterization of these atypical xenografts revealed them to be EBV-associated human B-cell lymphomas and discuss the implications of this observation.

## Results and Discussion

As summarized in Table 1, we obtained fresh HCC samples from 16 consecutive patients who were undergoing surgical resection of their tumors as primary therapy. None of the patients had received any form of neoadjuvant therapy. The diagnosis of HCC and degree of tumor differentiation in each resected specimen was determined through standard diagnostic assessments performed by clinical hepatopathologists at our institution independent of this study. Of 21 xenografts generated from the 16 different HCC specimens, we identified only 10 xenografts that resembled HCC on initial histopathological assessment (“HCC-like xenografts”). The remaining 11 xenografts resembled lymphoid neoplasms rather than HCC (“non-HCC-like xenografts”). In comparing the groups of patient samples that yielded HCC-like xenografts as compared with those that yielded non-HCC-like xenografts, there were no obvious differences between groups in patient age (68±11 years vs. 62±10 years, p = 0.27), underlying liver disease, or degree of differentiation of primary tumors. HCC-like and non-HCC-like xenografts arose in both NOD/SCID and NSG mice, and the time intervals between HCC implantation and harvesting of the first xenografts were similar (180±81 days vs. 144±65 days, p = 0.28).

**Table 1:** Patient demographics, parent HCC grade, and xenograft characteristics.

Xenograft Histology	Sample ID	Patient Age (yr)	Patient Sex	Liver Disease	HCC Grade^a^	Mouse Strain	Proportion of Mice with Xenografts		Days to Xenograft^b^
							From HCC Fragment	From HCC Cell Suspension	
**HCC-like**	51853	65	Male	Hepatitis C	Poor	NSG	–	2/4	215, 250 c
	55368	62	Male	NASH d	Moderate	NOD/SCID	1/4	–	64
	58063	81	Female	Hepatitis B	Poor	NOD/SCID	1/3	–	301
	59394	66	Male	Hepatitis C	Moderate	NOD/SCID	2/2	–	133, 209 c
	59410	50	Female	Hepatitis B	Moderate	NOD/SCID	0/2	1/1	120
	59826	69	Female	Alcohol	Moderate	NSG	2/2	0/2	85, 148 c
	60333	83	Female	Hepatitis B	Moderate	NSG	0/2	1/1	272
**Non-HCC-like** (**lymphoid**)	54069	72	Male	Cryptogenic	Moderate	NSG	1/6	–	140
	54307	60	Male	Hepatitis C	Moderate	NSG	2/2	–	100, 140 c
	55727	83	Female	Hepatitis B	Moderate	NOD/SCID	2/5	–	116, 193 c
	57602	61	Male	Hepatitis C	Moderate	NSG	1/3	0/2	85
	58424	60	Male	Hepatitis C	Poor	NOD/SCID	1/3	–	125
	59676	50	Male	Cryptogenic	Moderate	NSG	1/2	0/2	171
	59957	59	Male	NASH	Moderate	NSG	0/3	1/1	314
	60665	61	Male	Hepatitis B	Moderate	NSG	0/3	1/2	106
	62033	51	Male	Hepatitis B and NASH	Poor	NSG	1/4	–	92

a Degree of tumor differentiation documented in clinical pathology report.

b Number of days between implantation of tumor sample and harvesting of a 1.5 cm3 xenograft.

c Both xenografts demonstrated similar histology.

d Non-alcoholic steatohepatitis.

**Figure 1:**
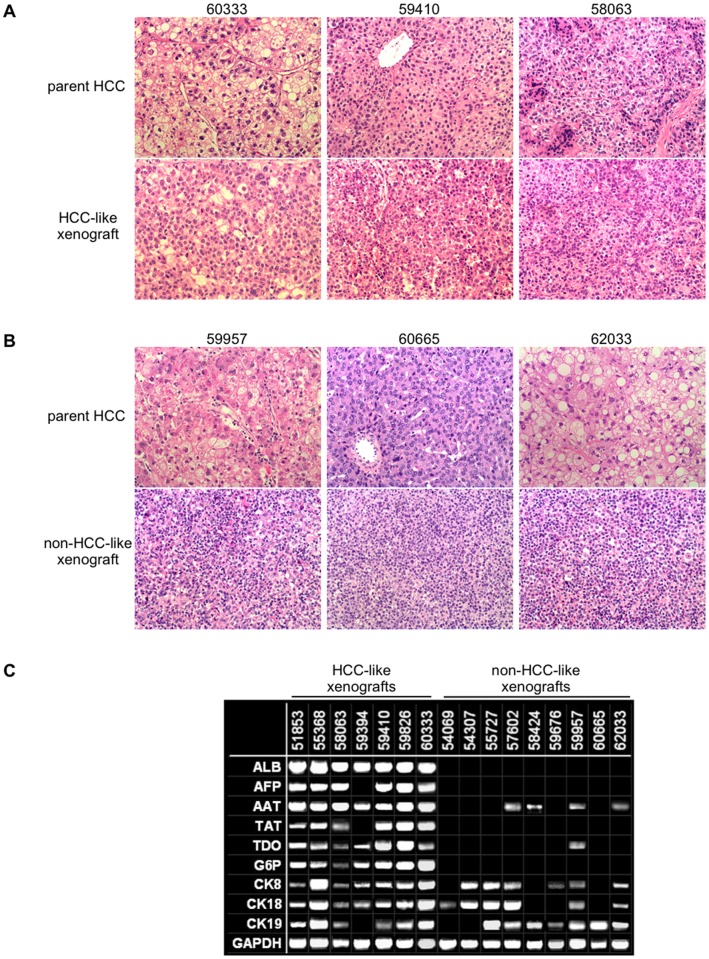
Xenografts arising from human HCC specimens. (A) Representative H&E sections (×200) of parent HCC tumors from three different patients (top panels) and the corresponding xenografts (bottom panels), which share typical histopathological features of HCC. (B) Representative H&E sections (×200) of parent HCC tumors from three different patients (top panels) that show typical features of HCC in contrast to the corresponding xenografts (bottom panels) which resemble lymphoid neoplasms. (C) RT-PCR demonstrating that xenografts which retain histopathological features of HCC (“HCC-like xenografts”) express typical liver cell markers, while many of these markers are absent from xenografts that do not resemble HCC histopathologically (“non-HCC-like xenografts”) (composite image; ALB – albumin, AFP – alphafetoprotein, AAT – alpha-1-antitrypsin, TAT – tyrosine aminotransferase, TDO – tryptophan-2,3-dioxygenase, G6P – glucose-6-phosphate dehydrogenase, CK8/18/19 – cytokeratin 8/18/19, GAPDH – glyceraldehyde phosphate dehdrogenase).

As shown in Figure 1A, HCC-like xenografts shared many histological features of HCC with parent tumors, including hepatocyte-like cells with nuclear atypia and high nuclear-to-cytoplasmic ratio, absence of portal tracts, and distorted trabeculae with increased thickness of hepatocellular plates. As shown in Figure 1B, non-HCC-like xenografts differed significantly from parent HCC tumors and HCC-like xenografts. Non-HCC-like xenografts had no architectural features of HCC, instead consisting of monomorphic populations of small lymphoid mononuclear cells with nuclear atypia and high mitotic index. As shown in Figure 1C, analysis of xenografts by RT-PCR revealed that HCC-like xenografts retained expression of many liver cell markers, while these were diminished or absent in non-HCC-like xenografts. We suspect that the persistent detection of liver epithelial markers such as cytokeratins, AAT and TDO in some non-HCC-like xenografts reflects the extreme sensitivity of RT-PCR to detect the presence of some persisting HCC cells in non-HCC-like xenografts despite near replacement of the originally implanted HCC tissue by rapidly proliferating lymphoid cells.

**Figure 2:**
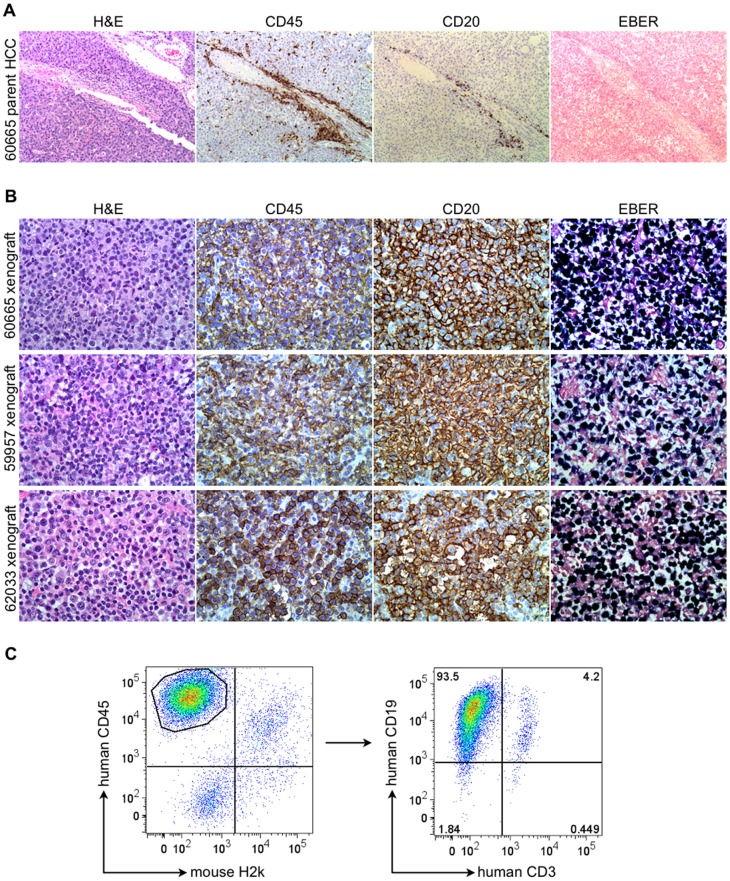
Expression of leukocyte markers and EBER in non-HCC-like xenografts. (A) Representative section (×200) from a parent HCC sample that gave rise to a non-HCC-like xenograft, demonstrating a typical distribution of CD45^+^ leukocytes along a portal tract invaded by the tumor, only a small fraction of which are CD20^+^ B lymphocytes; EBER ISH is negative. (B) Representative sections (×400) from three non-HCC-like xenografts demonstrating that a very high proportion of cells stain positively for human CD45 and human CD20 (brown), consistent with human B lymphocytes; EBER ISH is very strongly positive in the cells in these xenografts (dark blue). (C) Representative multiparameter flow cytometry analysis of freshly isolated cells from a non-HCC-like xenograft demonstrating that a large proportion of tumor cells are human CD45^+^ leukocytes (left plot), and that the majority of the gated CD45^+^ population also expresses human CD19^+^ (right plot), consistent with B lymphocytes.

Due to the resemblance of non-HCC-like xenografts to lymphoid neoplasms, we evaluated the expression of human and murine leukocyte markers by immunohistochemistry. In contrast to the typically sparse distribution of leukocytes observed in all of the parent human HCC tissues to a similar extent mainly along portal tracts when they were invaded by the tumor (Figure 2A), non-HCC-like xenografts were densely infiltrated or replaced by cells that could be characterized as human B lymphocytes by their expression of human CD45 and CD20 (Figure 2B). Flow cytometry confirmed that the human CD45^+^ population consisted predominantly of CD19^+^ cells, consistent with human B lymphocytes (Figure 2C). Staining for the human T-cell antigen CD3 was minimal, as was staining for the mouse lymphocyte antigen B220 and the mouse histocompatibility antigen H2k (data not shown). We did not identify any correlation between leukocyte infiltration in parent HCC tissues and the development of non-HCC-like xenografts. In addition, prior to their HCC resection, none of the source patients had a history of lymphoproliferative disease or an immunodeficient state known to predispose to lymphoproliferative disorders (eg. human immunodeficiency virus infection, pharmacological immunosuppression).

Cognizant of the importance of EBV in the pathogenesis of lymphoproliferative disorders in immunocompromised humans [18], we evaluated our xenografts for evidence of EBV infection by *in situ* hybridization (ISH) for EBV-encoded RNA (EBER), and found that this was strongly positive in the B lymphocytes populating the non-HCC-like xenografts (Figure 2B). We were unable to determine the presence or absence of latent EBV in parent HCC specimens using EBER ISH and PCR for EBV DNA, and this needs to be prospectively evaluated. Since EBV is so effectively suppressed in immunocompetent humans, we suspect that the tiny amount of EBER and EBV DNA that would be present in a few B-cells in the source tumor, of which we had extremely limited histological sections for retrospective analysis, was below the detection threshold of our assays. Preoperative EBV serology of source patients was not available since this is not routinely tested.

**Figure 3:**
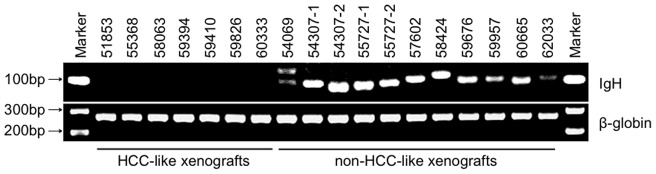
Immunoglobulin heavy chain gene rearrangements in non-HCC-like xenografts. PCR amplification of the variable region of the human IgH gene demonstrating unique dominant rearrangements in all of the non-HCC-like xenografts, confirming clonal B-cell proliferation. Dominant rearrangements were not amplified in HCC-like xenografts. Successful amplification of the β-globin gene confirms integrity of the genomic DNA analyzed.

To further characterize the non-HCC-like xenografts, we evaluated the clonality of the human B lymphocyte proliferations by assaying immunoglobulin heavy chain (IgH) gene rearrangements. As shown in Figure 3, B-cell proliferations arose from a single clone in 10 of 11 non-HCC-like xenografts, and from two clones in the remaining case. In two instances, independent xenografts obtained from separate fragments of a single parent HCC developed into lymphoid tumors that demonstrated different IgH rearrangements. This suggests that the malignant transformation and clonal expansion responsible for the EBV-associated lymphomas observed in this study occurred subsequent to the xenotransplantation of human tissue into immunodeficient mice, and that lymphomas were not already present in source tissues.

To exclude contamination by a lymphocyte cell line or cross-contamination between xenografts as a source of the lymphomas, we performed short tandem repeat (STR) analysis on genomic DNA from each xenograft and confirmed that xenografts from different patients were genetically distinct (Table 2).

**Table 2:** Short tandem repeat (STR) analysis demonstrating unique genetic identity of each xenograft.

Xenograft	Locus *															
	AMEL	CSF1PO	D13S317	D16S539	D18S51	D19S433	D21S11	D2S1338	D3S1358	D5S818	D7S820	D8S1179	FGA	THO1	TPOX	vWA
**51853**	XY	11,12	11,11	8,8	16,16	16,16	31.2,31.2	17,23	17,17	12,13	9,9	13,13	22,24	9.3,9.3	8,11	17,17
**55368**	XY	10,12	11,12	9,11	12,17	13,14	32.2,33.2	19,23	16,17	11,13	8,13	10,12	20,24	7,7	8,11	17,17
**58063**	XX	12,12	8,11	9,9	14,19	15.2,16,2	29,29	23,24	15,16	9,11	8,10	11,11	20.2,20.2	7,9	8,8	14,18
**59394**	XY	11,11	8,12	9,11	11,16	13,14.2	30,31	17,17	16,16	9,12	8,11	11,15	22,24	7,7	11,11	17,18
**59410**	XX	11,11	8,11	9,11	15,17	12,13	30,30	19,24	15,16	12,13	8,11	13,13	23,24	7,10	8,9	16,17
**59826**	XX	10,10	8,8	11,11	19,19	14,15	30,31.2	18,18	14,14	11,13	9,9	11,11	21,21	9,9.3	8,9	17,19
**60333**	XX	10,11	11,12	12,13	13,15	12,14	27,29	17,20	15,18	11,12	8,13	10,11	18,21	7,7	8,11	15,17
**54069**	XY	11,13	8,11	8,10	13,16	13,14.2	30.2,32.2	17,25	16,17	11,12	7,11	10,11	20,25	6,9	8,9	17,20
**54307**	XY	10,13	9,12	11,11	12,12	14,15	29,30	17,25	15,17	11,13	8,10	13,14	20,23	8,9.3	8,8	16,17
**55727**	XX	10,11	8,11	9,10	16,17	14,15	30,32	23,24	15,16	10,11	11,11	10,13	19,21	7,9	11,11	14,16
**57602**	XY	7,9	8,12	10,12	12,16	14,14	28,30	17,18	16,16	11,12	10,12	14,15	26,26	7,9	9,10	15,17
**58424**	XY	–	10,12	12,13	15,15	15,15.2	32.2,33.2	–	15,16	9.12	–	16,17	22,25	6,7	8,11	14,18
**59676**	XY	10,10	9,11	11,13	14,16	12,16	30,30	20,20	15,16	12,12	8,10	12,13	21,21	9.3,9.3	8,10	17,18
**59957**	XY	12,12	8,13	8,11	15,19	14,15.2	29,29	17,20	16,17	12,14	8,10	10,14	20,25.2	7,9.3	8,11	14,17
**60665**	XY	12,12	7,11	9,10	13,13	13,16.2	31,32.2	18,25	15,15	10,11	11,11	11,13	22,23	7,9	8,10	14,16
**62033**	XY	12,13	8,8	9,9	11,14	13,14.2	30,32	18,23	14,16	7,14	10,11	11,15	22,23	7,7	8,8	14,14

* Number of repeats on each chromosome at each of 15 tetranucleotide repeat loci for each xenograft are shown along with patient sex determination by amplification of the Amelogenin gender determining marker (AMEL).

Collectively, these findings demonstrate that non-HCC-like xenografts were comprised of EBV-associated human B-cell lymphomas that had developed spontaneously from xenografted human HCC tissues. Analogous to EBV-associated lymphoproliferative disorders in immunocompromised humans, this likely occurred through reactivation of latent EBV and malignant transformation of intratumoral passenger B lymphocytes made possible by the immunodeficiency of the recipient mice. In future work, we plan to apply cell sorting techniques to exclude CD45^+^ cells from human HCC samples prior to xenotransplantation in order to prevent this process.

The phenomenon of EBV-associated lymphomagenesis has not been reported in the context of human solid tumor xenografts. Considering the high prevalence of latent EBV in humans [16] and the presence of B lymphocytes in solid tumors from all human tissues [22], it is likely that most solid tumors would be vulnerable to this process. It is unlikely that this phenomenon is unique to HCC, as EBV has not been implicated in the pathogenesis of human HCC [23], primary hepatic lymphoid malignancies are very rare [24], and there is no clearly established causative link between chronic liver disease and the incidence of lymphoproliferative disease. We thus believe that our observations are of importance to all investigators utilizing human solid tumor xenotransplantation assays. As we have shown, lymphomas can arise within the first xenografts obtained, and do not require serial tumor passaging. Unrecognized, this process may confound experimental data such as measures of tumor growth and markers of tumor cell populations. Lymphomas may also competitively eliminate the tumor tissue of interest, resulting in loss of valuable samples.

This process can best be recognized through phenotyping of xenografted tissues using leukocyte markers. Histopathological assessment is not sufficient, as lymphomas may be misinterpreted as poorly differentiated epithelial tumors. Initial growth kinetics of lymphomas in our model was similar to HCC-like xenografts, indicating that this process cannot be detected by gross observations alone. Because lymphomas may arise from one or two B-cell clones, immunomagnetic depletion of CD45^+^ cells may not be sufficient to prevent this process since all CD45^+^ cells are not removed [25]. Flow cytometry-based single-cell sorting is the most stringent technique to identify and eliminate human leukocytes from solid tumor xenotransplantation assays. Most rigorous analyses of human solid cancer xenografts incorporate routine quantification, exclusion or depletion of contaminating CD45^+^ cells from assays targeting biological and functional properties of specific subpopulations of tumor cells [26]; our observations provide a compelling biological rationale to support this practice and extend it to include all observations involving solid tumor xenografts.

It is important to note that the generalizability of our observations should be interpreted with caution in the context of a relatively small sample size of patients, HCC specimens, and xenografts. The clinical characteristics of the source patients are observational only and were retrospectively analyzed, making this study vulnerable to potentially confounding patient factors or differences that it is underpowered to detect. Similarly, although our data does not demonstrate any obvious relationships between the xenografting methods used (eg. tumor fragment vs. cell suspension, NSG vs. NOD/SCID mouse strain) and the development of EBV-associated lymphomas, the study is underpowered to detect the relative influence of these variables on the results.

In summary, we have shown that human solid tumor xenografts in immunodeficient mice are vulnerable to lymphomagenesis associated with EBV. This potentially confounding process can be recognized through immunophenotyping of xenografts using leukocyte markers and should be preventable by excluding leukocytes from source tissues. This phenomenon should be recognized as an important pitfall of human solid tumor xenotransplantation assays.

## Materials and Methods

### Ethics statement

Ethical approvals were obtained from the University Health Network Research Ethics Board (Protocol #08-0697-TE) and Animal Care Committee (Animal Use Protocol #1595). Human tissues were obtained with written consent from source patients.

### Patient samples

Human HCC samples and demographic data were obtained from patients undergoing surgery and anonymized with 5-digit numbers. Tumor samples were obtained only from patients for whom surgical resection was the primary form of HCC treatment, and who had not received any form of systemic or locoregional neoadjuvant therapy (eg. systemic chemotherapy, intrahepatic chemotherapy, radiofrequency tumor ablation, external beam radiation therapy). Fresh surgical resection specimens were transferred from the operating room to the surgical pathology suite within 15 minutes of removal from the patient and promptly sectioned by the attending clinical pathologist. Samples for xenografting were taken from the peripheral zone of the tumor in all cases, and appeared grossly viable and non-necrotic. Samples were placed in serum-free Dulbecco's Modified Eagle Medium (DMEM, Life Technologies) at 4 degrees Celsius and transferred to the research laboratory for immediate processing and implantation into recipient mice. HCC diagnoses were subsequently verified in all cases using clinical pathology reports issued independent of this study.

### Xenografts

Bulk tumor cell suspensions were prepared by digestion with Type IV Collagenase (Sigma) for 30–60 minutes at 37°C, passage through a 70 µm cell strainer (BD Biosciences) and lysis of red blood cells using RBC lysis buffer (eBioscience). Following trypan blue analysis, 106 viable cells were resuspended in 50 µl of Matrigel (BD Biosciences) and injected subcutaneously with a 25 gauge needle into the flanks of anesthetized NOD/SCID or NSG mice. No attempt was made to separate HCC cells from other cell populations within tumor samples. Alternatively, 3 mm3 tumor fragments in Matrigel were implanted subcutaneously on the flanks through a 5 mm skin incision. Priority was given to implantation of tumor fragments; bulk cell suspensions were injected if sufficient tumor tissue was available and if the fraction of viable cells in the tumor cell suspension exceeded 70%.

### RT-PCR

RNA was isolated using TRIZOL Reagent (Life Technologies) and reverse-transcribed using SuperScript First-Strand Synthesis System (Invitrogen). PCR was performed with Taq DNA Polymerase (New England Biolabs) using conventional thermocycling protocols. Amplified products were visualized with ethidium bromide agarose gel electrophoresis. Primer sets were synthesized based on publicly available human nucleotide sequences (see Table S1).

### Histopathology

Formalin-fixed, paraffin-embedded tissues were utilized. For immunohistochemistry, de-waxed sections were blocked with 3% hydrogen peroxide, avidin/biotin blocking kit (Vector Labs), and 10% normal serum from the secondary Ab species, then incubated at room temperature with primary Ab for 1 hour as follows: mouse anti-human CD45 (1∶80, Dako), mouse anti-human CD20 (1∶100, Dako), rabbit anti-human CD3 (1∶300, Dako), or rat anti-mouse B220 (1∶1000, BD Biosciences). This was followed by biotin labeled secondary Ab (Vector Labs) for 30 minutes and HRP-conjugated ultrastreptavidin labeling reagent (ID Labs) for 30 minutes. Color was developed with DAB solution (Dako). Sections were counterstained with Mayer's hematoxylin, dehydrated and mounted in Permount (Fisher). *In situ* hybridization for EBER was performed on a Ventana Medical Systems automated slide stainer employing EBER probes and the Ventana ISH iVIEW Blue Detection Kit according to manufacturer instructions.

### Flow cytometry

Cells were blocked in PBS containing 0.5% BSA and human FcR Blocking Reagent (Miltenyi Biotec), then incubated with anti-mouse H2-K [d] FITC (SF1-1.1, BD Biosciences), anti-human CD45 PE-Cy7 (HI30, BD Biosciences), anti-human CD3 Alexa Fluor 647 (UCHT1, BioLegend) and anti-human CD19 PE (HIB19, BioLegend), or with FITC Mouse IgG2a, κ (G155-178, BD Biosciences), PE-Cy7 Mouse IgG1, κ (MOPC-21, BD Biosciences), Alexa Fluor 647 Mouse IgG1, κ (MOPC-21, BD Biosciences) and PE Mouse IgG1, κ (MOPC-21, BD Biosciences) isotype controls. After washing, cells were incubated with Live/Dead Fixable Violet Dead Cell Stain Kit (Invitrogen) and analyzed using LSR II Flow Cytometer (BD Biosciences) with FlowJo V8.8.6.

### IgH gene rearrangement assay

DNA was isolated using GenElute Mammalian Genomic DNA Miniprep Kit (Sigma). Clonality was evaluated by PCR for VDJ rearrangement of the IgH gene using primers directed at framework three of the V segments (IgHV: ACACGGCC(A/C/G)TGTATTACTGT) and J segments (IgHJ: TGAGGAGACGGTGACC), flanking the hypervariable gene region. PCR was performed with Taq DNA Polymerase (New England Biolabs) and conventional thermocycling protocols. Amplified fragments were visualized with ethidium bromide agarose gel electrophoresis. Clonality was determined by the number of dominant bands, and bands of unique size were interpreted as products of distinct B-cell clones.

### STR analysis

Genotyping was performed using the AmpFlSTR Identifiler PCR Amplification Kit (Life Technologies) that employs a multiplex assay which amplifies 15 tetranucleotide repeat loci and the Amelogenin gender determining marker. Samples were run on an ABI 3100 Genetic Analyzer and analyzed in GeneMapper v3.7.

### Statistical analysis

Where applicable, unpaired t-tests were used to compare means between two groups and statistical significance was expressed with two-tailed P values.

## Supporting Information

Table S1 PCR primer sets.
(DOC)
