# Supplementary material for: Human Solid Tumor Xenografts in Immunodeficient Mice Are Vulnerable to Lymphomagenesis Associated with Epstein-Barr Virus
Source: PLoS One. 2012 Jun 18;7(6):e39294. doi: 10.1371/journal.pone.0039294 (PMC3377749; doi:10.1371/journal.pone.0039294)
Supplement: Table S1 — PCR primer sets. (DOC) [file pone.0039294.s001.doc]

**Table S1. PCR primer sets**

| **Gene*** | **Accession** | **Forward Primer (5’-3’)** | **Reverse Primer (5’-3’)** | **Annealing Temp (°C)** | **Product (bases)** |
| --- | --- | --- | --- | --- | --- |
| **ALB** | NM_000477 | ACAAAGATGACAACCCAAACC | ATCCTTTGCCTCAGCATAGTT | 52.8 | 659 |
| **AFP** | NM_001134 | TCTTCTTTGGGCTGCTCGCTAT | CAATAACTCCTGGTATCCTTTAGCAACT | 53.2 | 610 |
| **AAT** | NM_000295 | GGACCAAGGCTGACACTCACGATG | TGGACAGTTTGGGTAAATGTAAGC | 55.9 | 678 |
| **TAT** | NM_000353 | CCATCCATCGGCTTCCTATCC | GTTTGCTGAACACTGACCCACA | 54.1 | 352 |
| **TDO** | NM_005651 | CTGGGAGTTGGATTCTGTTCG | GACAGCCGTCTTTCACCTTTACT | 53.3 | 594 |
| **G6P** | NM_000151 | GACCTACAGATTTCGGTGCTTGA | GAGGACGAGGGAGGCTACAATA | 55.7 | 484 |
| **CK8** | NM_002273 | CCGCAGTTACGGTCAACCAGAG | TCGTCGGTCAGCCCTTCCAG | 57.7 | 469 |
| **CK18** | NM_000224 | GGTCTGGCAGGAATGGGAGG | TGGCAATCTGGGCTTGTAGGC | 57.3 | 481 |
| **CK19** | NM_002276 | TGCTGGCGGGCAACGAGAAG | CCTGGATGGTCGTGTAGTAGTGGCTGT | 61.1 | 183 |
| **GAPDH** | NM_002046 | GCGCTGAGTACGTCGTGGAGT | AAGGTGGAGGAGTGGGTGTCG | 58.8 | 613 |

* Gene abbreviations: ALB – albumin, AFP - alphafetoprotein, AAT - alpha-1-antitrypsin, TAT - tyrosine aminotransferase, TDO - tryptophan-2,3-dioxygenase, G6P - glucose-6-phosphate dehydrogenase, CK8/18/19 - cytokeratin 8/18/19, GAPDH - glyceraldehyde phosphate dehdrogenase)
